# Nephrotoxicity of Calcineurin Inhibitors in Kidney Epithelial Cells is Independent of NFAT Signaling

**DOI:** 10.3389/fphar.2021.789080

**Published:** 2022-01-24

**Authors:** Andrea Karolin, Geneviève Escher, Stefan Rudloff, Daniel Sidler

**Affiliations:** ^1^ Department for Nephrology and Hypertension, University Hospital Insel Bern, Bern, Switzerland; ^2^ Graduate School for Cellular and Biomedical Sciences, University of Bern, Bern, Switzerland

**Keywords:** calcineurin inhibitor, nephrotoxicity, NFAT-independent, p38 kinase, PI3K - AKT

## Abstract

**Background:** Calcineurin inhibitors (CNIs) such as cyclosporine A and tacrolimus are commonly used after renal transplantation to suppress the immune system. In lymphoid cells, cyclosporine A acts *via* the calcineurin/nuclear factor of activated T-cell (NFAT) axis. In non-lymphoid cells, such as kidney epithelial cells, cyclosporine A induces calcineurin inhibitor toxicity. It is unknown *via* which off-targets cyclosporine A induces calcineurin inhibitor toxicity in kidney epithelial cells.

**Methods:** To measure a compound’s potential to induce nephrotoxicity, the expression of the surrogate marker Fn14 was measured by flow cytometry. Compounds were tested for their potential to induce Fn14 either chemically or plasmid-mediated. Mice were injected with various compounds, and changes in nephrotoxic gene expression levels of the kidney epithelial cells were then analyzed.

**Results:** Fn14 is specifically upregulated due to calcineurin inhibitor toxicity inducing agents. Inhibition of the NFAT axis showed no increase of the Fn14 expression on the surface of kidney cells. However, inhibition of p38 MAPK, phosphoinositide-3-kinase (PI3K)/Akt, protein kinase C (PKC), and inhibitor of nuclear factor-*κ*B (I*κ*B) kinase (IKK) showed clear induction of Fn14 and increased expressions of nephrotoxic, inflammatory, and fibrotic genes *in vitro* and *in vivo*.

**Conclusions:** These findings show that cyclosporine A acts independently of NFAT on kidney epithelial cells. Moreover, inhibition of serine/threonine protein kinases mimics cyclosporine A’s activity on kidney epithelial cells. This mimicking effect indicates that these protein kinases are off-targets of cyclosporine A and damage structural renal cells when inhibited and therefore contributes likely to the development and progression of calcineurin inhibitor toxicity.

## Introduction

Calcineurin inhibitors (CNIs), e.g., cyclosporine A (CsA) and tacrolimus (FK506), are pivotal drugs for the prevention of rejection after solid organ transplantation. CNIs show a highly interindividual pharmacokinetic profile and have a narrow therapeutic range. Even mild overdosing may lead to substantial acute and chronic side effects, including tumor development, infections, metabolic disturbances, and kidney failure. Calcineurin inhibitors induce nephrotoxicity through (reversible) vasoconstriction in the vas afferent. Vasoconstriction leads to consecutive relative hypoxia and progressive athero- and arteriolohyalinosis, tubular atrophy, and interstitial fibrosis (Feske and Vaeth, 2018). Recent register studies have demonstrated that virtually all kidney transplanted patients develop signs of chronic calcineurin inhibitor toxicity (CNT) within 10 years after kidney transplantation (Stegall et al., 2018; Karolin et al., 2021). In lymphocytes, CsA inhibits the calcineurin/nuclear factor of activated T-cell (NFAT) axis leading to repression of transcriptional programs necessary for activation, proliferation, and cytokine production. This inhibition primarily contributes to the immunosuppressive effect of CsA (Kiani et al., 2000; Casey and Meier-Kriesche, 2011).

Members of the TNF superfamily have a non-redundant role in the pathogenesis of tissue regeneration and wound healing but are also critically involved in chronic inflammatory and fibrosis (Croft, 2009; Burkly, 2015; Croft and Siegel, 2017). We have recently shown that the tumor necrosis factor (TNF)–related weak inducer of apoptosis (TWEAK) is indispensable for developing CNT in mice (Claus et al., 2018). CsA induces the expression of TWEAK’s receptor, fibroblast growth factor–inducible 14 (Fn14), in kidney epithelial cells, which facilitates inflammatory and fibrotic signals critical for progressive nephrotoxicity. Deficiency for TWEAK or treatment with TWEAK-neutralizing reagents protected animals from acute CNT lesions (Zhao et al., 2007; Sanz et al., 2008; Xia et al., 2015; Claus et al., 2018). Furthermore, administration of recombinant TWEAK (rTWEAK) induced similar disease as when animals are treated with CsA alone. Interestingly, the combination of rTWEAK and CsA caused severe tubulopathy and interstitial inflammation (Claus et al., 2018). The receptor Fn14 is low ubiquitously expressed on epithelial cells and can be induced upon stress and injury stimuli (Burkly, 2014). Fn14 is the only known receptor to the cytokine TWEAK (Bossen et al., 2006). TWEAK is produced by infiltrating and tissue-resident immune cells, largely monocytes and neutrophils (Campbell et al., 2006; Vince and Silke, 2006; Winkles, 2008).

Currently, it is unclear how the CNI, CsA, interferes with kidney epithelial cells and induces nephrotoxicity. The present work aims to investigate off-targets of CsA in renal epithelial cells. We hypothesize that CsA elicits inflammatory and fibrotic activities in renal epithelial cells independent of NFAT but *via* inhibition of kinase pathways.

## Materials and Methods

### Cell Culture Experiments

The murine kidney epithelial cell line (MCT) has been described previously (Haverty et al., 1988). Cells were grown in 10% fetal calf serum (FBS) supplemented with Dulbecco modified Eagle medium (DMEM; ThermoFisher, 11965092) and sub-cultivated in a 12-well format with 50,000 cells per well. HEK293T cells were cultured in DMEM/10%FBS and 2 mM L-glutamin in a 6-well (800,000 cells/well) and 12-well (300,000 cells/well) format. The next day, cells were stimulated for 24 h with either 10 μg/ml CsA (Sandimmun Neoral^®^ from Novartis) or 10 μg/ml FK506 (Cell Signaling, Daveres, MA). In some experiments, cells were incubated for 24 h with the following reagents: 1 μM 11R-VIVIT (NFAT Inhibitor) (Merck, 480401), 10 μM SB203580 (Sigma, S8307), 10 μM SB202190 (Sigma, S7076), gentamicin, amphotericin B, 10 μg/ml cisplatin (Sigma, C2210000), or rTWEAK (PeproTech, 310-06). HEK293T cells were transfected with 2.5 μg of plasmid DNA using standard lipofectamine 3,000 protocols (ThermoFisher, L3000001). The transfected plasmids were pCMV-VIVIT-GFP (Addgene, 11106), pCMV-p38-CA-EGFP, and pCMV-eGFP-N1 (Addgene, 6,085-1). Following transfection, various treatments were performed the next day for 24 h. For the top-down kinase inhibitor screen, MCTs were cultured in a 12-well format, as described above. The next day, cells were treated with various kinase inhibitors (Enzo, BML-2832) at 10 μM for 48 h and then analyzed by flow cytometry (described below).

### Acute Toxicity Mouse Model

As previously described (Claus et al., 2018), acute CNT lesions were induced by injecting (intraperitoneal) animals with 100 mg/kg CsA (Sandimmun Neoral^®^), 10 mg/kg p38 mitogen-activated protein kinase (MAPK) inhibitor (SB203580, SB202190), and 1 mg/kg 11R-VIVIT on three consecutive days twice daily. The dosage of CsA was chosen based on the publication from Claus et al., 2018. Concentrations for the p38 MAPK inhibitor and 11R-VIVIT were based on previously published *in vivo* studies, where these compounds showed efficacy at indicated concentrations (Seeger et al., 2010; Zhang et al., 2013). Animals were then euthanized, and kidneys and blood were collected for subsequent analysis. Red cell lysis buffer (Roche, 11814389001) was added to the blood cells until all erythrocytes were removed. The white blood cells were used for flow analysis (Described below). Mouse kidneys were digested with 2 mg/ml collagenase I (Sigma, C9891) in 1% BSA in PBS together with DNase for 20min at 37°C. The cell suspension was filtered through a cell strainer, washed with 1%BSA in PBS, and centrifuged at 200xg for 3min in 15 ml total volume. The cell suspension was then stained with antibodies for flow cytometry (described below).

### Antibody Staining

Flow cytometry was performed on dissociated MCT or HEK293T cells stained with the Fn14-APC antibody (1:30; Miltenyi, 130-104-281). For negative control, unstained cells or the respective isotype control antibody, mouse IgG2b (1:100, Miltenyi, 130-098-890) was used. Antibody staining was conducted for 30 min in the dark at 4°C. Proliferation assay was conducted as follows: from naïve animal’s lymph node/Spleen, T cells were isolated and stained with 1:1000 e450 proliferation dye (ThermoFisher, 65-0842-85) and treated with various reagents (11R-VIVIT, CsA). Cells were then stimulated with ⍺CD3/⍺CD28 dynabeads for 5 days. White blood cells were stained for 30 min in the dark at 4°C with CD45-PerCP/Cy5-5 (1:100; Biolegend, 103132) and CD11b-APC/Cy7 (1:100; Biolegend, 101226). The kidney cell suspension was stained with CD45-PerCP/Cy5-5 and CD326-PE (1:50, Miltenyi, 130-117-667) for 30min in the dark at 4°C. Also, single stains were created to perform proper compensation.

### Flow Cytometer and Cell Sort

LSR-II flow cytometer, with the software FACS Diva, was used to determine the Fn14 surface expression, proliferation assay, and blood cell flow analysis. The geometric mean fluorescence intensity (MFI) of antibody staining or total count of cells was calculated by FlowJo Software. Most of the figures showing the Fn14 expression are based on measurements conducted by flow cytometry.

The flow facility at the University of Bern sorted mouse kidney cells. The sort was conducted on the MoFlo Astrios EQ instrument. After DAPI (1:100′000) was added, gating was conducted on DAPI-negative, CD45-negative, and CD326-positive cells. Living CD45-/CD326 + cells were collected in FBS.

### RNA Isolation, Reverse Transcription, and RT-qPCR

Total RNA was isolated from samples using TRIzol reagent (Invitrogen, 15596026). A Nanodrop 1000 spectrophotometer was used to measure the RNA concentration and evaluate its quality. A PrimeScript RT Reagent Kit (Takara, RR037A) was used to generate from 1 μg RNA cDNA. cDNA was diluted to 2 ng/μl. An RT^2^ Profiler PCR Array for mouse nephrotoxicity (PAMM-094Z) was used for determining the expression of nephrotoxic genes. According to the manufacturer’s protocol, the RT^2^ Profiler PCR Array analysis was conducted with SYBR green (Qiagen, 330520). The relative gene expression was calculated with the delta–delta Ct method (2^^−ΔΔCt^). Normalization was conducted with provided housekeeping genes on the array.

### RNAseq

To the sorted cells from mouse kidneys, TRIzol was added. Total RNA extraction was conducted, as described above. RNA sequencing was performed on Illumina NovaSeq600 with a TruSeq stranded mRNA library. The Bioinformatics facility then normalized the RNAseq data at the University of Bern. First, RNAseq data quality was determined, and reads were mapped to the reference genome. Then, gene expression differences between the various treatment groups were identified. Normalized data were then visualized by using the heatmap tool on Morpheus.

### Protein Isolation and Analysis

Cells were lysed in Laemmli buffer and separated by a 12% sodium dodecyl sulfate–polyacrylamide gel electrophoresis. Then, transfer on a polyvinylidene fluoride membrane (ThermoFisher, 88518) was conducted. Membranes were blocked with 5% milk in Tris-buffered saline with Tween 20 (TBST) for 1 hour. Incubation with the primary antibody against Fn14 (1:1000; Cell Signaling, 4403S) and GAPDH (1:50′000; Merck, cB1001) was conducted overnight at 4°C. The horseradish peroxidase–conjugated secondary antibody was added to the membrane for 1 hour at room temperature (1:5000, Jackson, 711-035-152 and 1:5000, Jackson, 715-035-151). Visualization of the proteins was conducted using SuperSignal (ThermoFisher, 34076).

### Statistical Analysis

Analysis was performed using Prism 5 software (GraphPad, San Diego, CA). Results are the mean ± the standard deviation (SD). Groups were analyzed by Student’s t-test (unpaired) or ordinary one-way ANOVA and Tukey’s multiple comparison test, with a single pooled variance. *p* values below 0.05 were considered significant (ns: *p* > 0.05, *: *p* ≤ 0.05, **: *p* ≤ 0.01, ***: *p* ≤ 0.001, and ****: *p* ≤ 0.0001).

## Results

### Surface Fn14 Is Induced Upon CsA Exposure in Kidney Epithelial Cells but Not in Response to the Cell-Permeable NFAT Inhibitor

**FIGURE 1:**
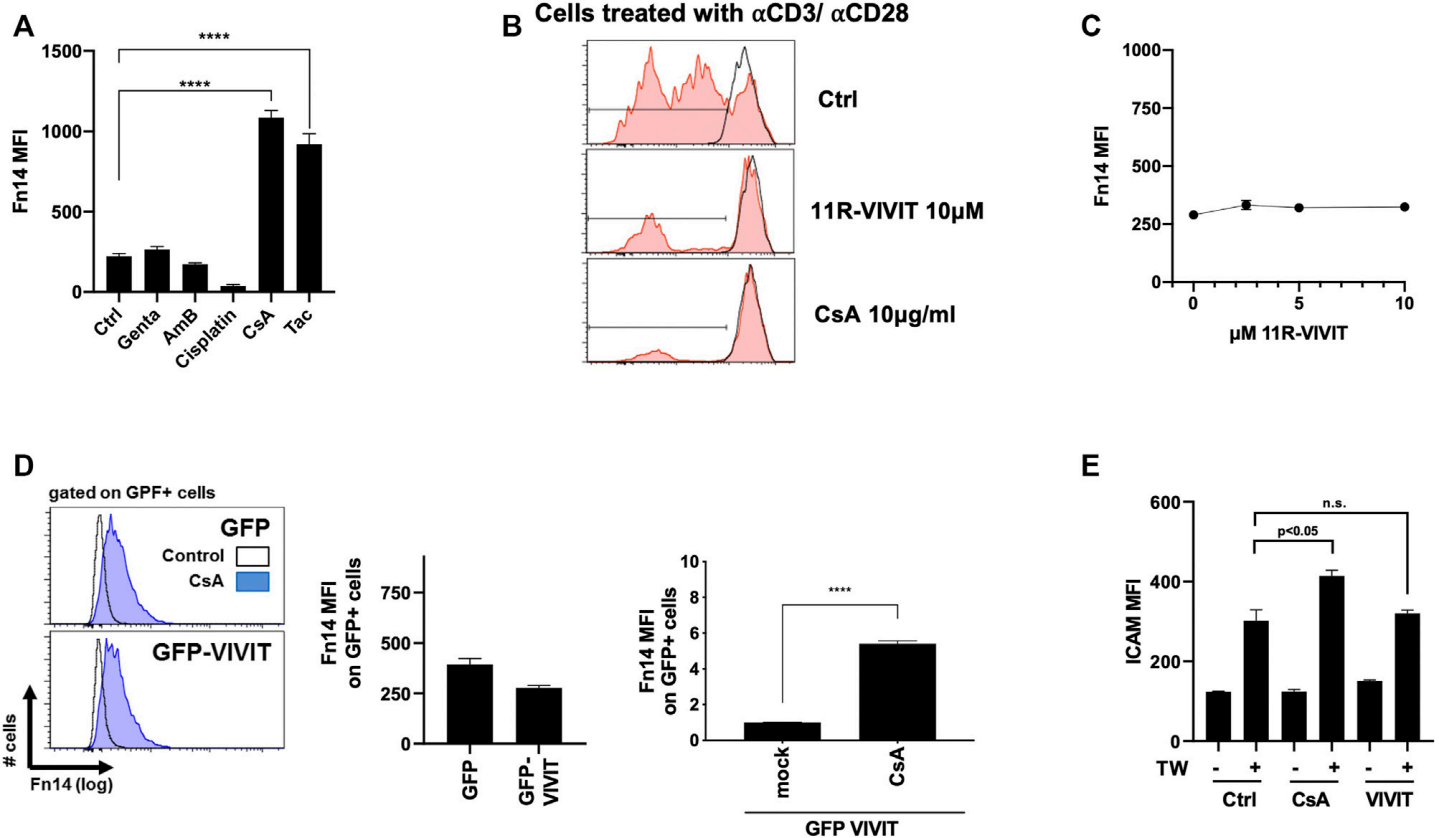
Fn14 induction upon CsA exposure but not upon NFAT inhibition. **(A)** Fn14 induction with CsA and Tac (CNIs) but not with gentamicin, amphotericin B, and cisplatin (MCT). **(B)** NFAT and CsA successfully inhibit proliferation of TCRab-stimulated T cells *ex vivo*. **(C-D)** Inhibition of the NFAT axis, by plasmid chemically (11R-VIVIT) or (GFP VIVIT), shows no upregulation of Fn14 on the cell surface (C: MCT, D: HEK293T). **(E)** Cells sensitized with CsA show upregulation of ICAM upon TWEAK treatment. Inhibition of NFAT combined with TWEAK treatment shows reduced ICAM expression, comparable to control conditions (MCT). For all figures, flow cytometry was used.

We have previously demonstrated that the calcineurin inhibitor CsA induces the Fn14 surface expression on kidney epithelial cells *in vitro* and *in vivo* (Claus et al., 2018). To further support these findings, we challenged MCT cells with various nephrotoxins. Strikingly, FK506, similar to CsA, induced a Fn14 threefold in MCT cells, while gentamicin, amphotericin B, and cisplatin did not do so (Figure 1A). As CsA inhibits the NFAT pathway in lymphoid cells, we blunted the NFAT activity in kidney epithelial cells by treatment with direct NFAT inhibitors, namely, either cell-permeable 11R-VIVIT or plasmid-mediated transient expression of the VIVIT plasmid. Chemical 11R-VIVIT was tested on TCR-antibody–stimulated T cells *ex vivo* (Figure 1B). T cells treated with 11R-VIVIT or CsA showed no distinct peaks indicating no new cell generation compared to control cells. Meanwhile, treatment of MCT cells with equal doses of 11R-VIVIT or transient transfection of HEK293T cells with the VIVIT plasmid did not increase the Fn14 expression *in vitro* (Figures 1C,D). Also, CsA induced further upregulation of Fn14 on cell surfaces, and even the NFAT axis was inhibited (plasmid-mediated) (Figure 1D). It has been shown that CsA sensitizes cells to the cytokine TWEAK (Claus et al., 2018). We show in our experiments how cells express significantly higher the adhesion molecule ICAM after TWEAK treatment when pre-sensitized with CsA. This pre-sensitization is no longer observed when NFAT is inhibited in these cells, comparable to control (Figure 1E).

### Inhibitors of Protein Kinases Mimic Effects of CsA in Kidney Epithelial Cells

**FIGURE 2:**
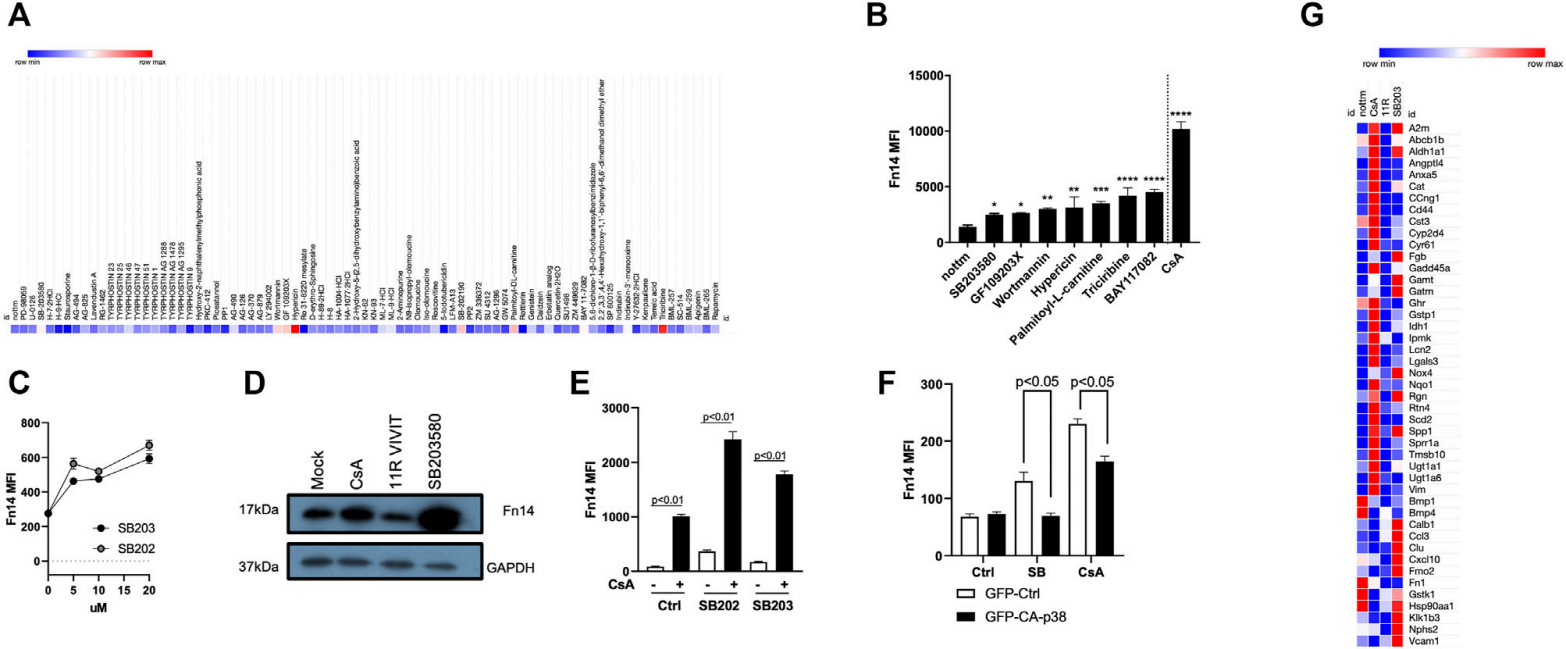
Effects of CsA on kidney epithelial cells are mimicked by protein kinase inhibitors. **(A-B)** Protein kinase library screen revealed that inhibition of PI3K/Akt, IKK, and p38 MAPK induces Fn14 (MCT). **(C)** Titration of two p38 MAPK inhibitors (SB203580 and SB202190) revealed the similar potential to induce Fn14 (MCT). **(D)** Protein levels of Fn14 are increased upon CsA and SB203580 treatment but not when NFAT is inhibited (MCT, Western Blot). **(E)** Increased expression of Fn14 is detected when cells are treated simultaneously with the p38 MAPK inhibitor and CsA compared to single treatment with inhibitors (MCT cells). **(F)** Constitutive active p38 MAPK shows reduced Fn14 expression on cells when treated with either SB203580 or CsA (HEK293T). **(G)** Nephrotoxic genes are upregulated with CsA and with SB203580 (MCT, RT-PCR). If not indicated differently, results were acquired by flow cytometry.

To investigate CsA’s effect in tubule epithelial cells of the kidneys, we treated MCT cells with the kinase inhibitor library. We investigated the effect of individual compounds on Fn14 induction as a surrogate marker for pro-fibrotic responses. Indeed, only specific protein kinase inhibitors (Figures 2A,B) significantly upregulated Fn14 on the surface of MCT cells. Interestingly, inhibition of calmodulin kinase II (by KN-62 and KN-93) showed no upregulation of Fn14 (Figure 2A). Many inhibitors, such as BAY 11-7082, SB203580, wortmannin, GF 109203X, palmitoyl-dl-carnitine, triciribine, SB202190, and hypericin led to significant induction of surface Fn14. Notably, two p38 MAPK inhibitors (SB203580 and SB202190) showed the potential to upregulate Fn14 on cell surfaces. Titration of both inhibitors revealed a similar potential to induce Fn14 (Figure 2C). Protein from SB203580-treated cells showed similar upregulation of Fn14 as cells treated with CsA (Figure 2D). Cells treated simultaneously with a p38 MAPK inhibitor and CsA showed an increased expression of Fn14 compared to cells only treated with CsA or one of the p38 MAPK inhibitors (Figure 2E). To corroborate these findings, we generated cells expressing a constitutive active p38 MAPK (pCMV-p38-CA-EGFP). The basal Fn14 expression (MFI) was moderate in all untreated mock-transfected (pCMV-eGFP-N1) and p38-CA–expressing cells. Mock-transfected control (GFP) showed a highly significant upregulation of Fn14 upon SB203580 or CsA stimulation. Meanwhile, the p38-CA–expressing cells treated with the p38 MAPK inhibitor or CsA induced a significantly lower Fn14 expression (Figure 2F). Furthermore, a gene array revealed that CsA and SB203580 upregulate various genes, whose expression levels did not alter when 11R-VIVIT was used. Nphs2, Cat, Rgn, A2m, Fgb, Ccl3, Cxcl10, Vcam1, and Nox4 and Spp1 are such upregulated genes (Figure 2G). Disorders in Nphs2, Cat, and Rgn genes are associated with kidney diseases (Franceschini et al., 2006; Ghaly et al., 2012; Yamaguchi, 2015). Upregulated genes such as Fgb, Ccl3, Cxcl10, Cd44, and Vcam are known to be involved in inflammatory responses (Nguyen and Simpson-Haidaris, 2000; Lim and Luscinskas, 2006; Johnson and Ruffell, 2009; Bhavsar et al., 2015; Lee et al., 2017; Kong et al., 2018). Lastly, also genes indicating the onset of fibrosis (Nox4 and Spp1) are upregulated (Amara et al., 2010; Morse et al., 2019).

### CsA, but Not VIVIT, Induces Nephrotoxicity *in vivo*

**FIGURE 3:**
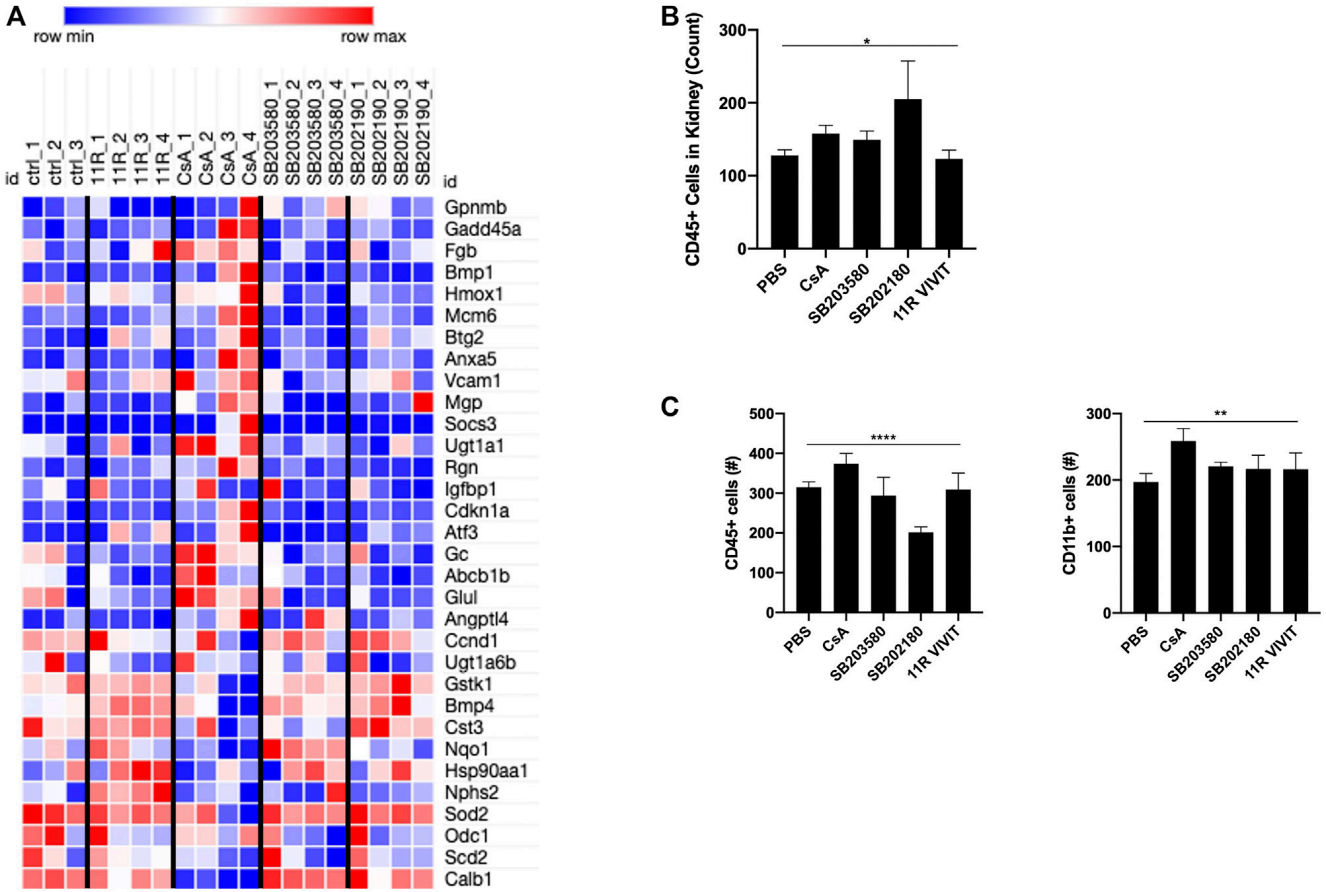
*In vivo* experiments show the development of nephrotoxicity upon CsA and p38-kinase inhibitor. **(A)** Nephrotoxic gene array reveals evident upregulation of genes involved in nephrotoxicity upon CsA treatment. Also, both p38 inhibitors revealed upregulation of nephrotoxic genes (mouse kidney cells, RNAseq). **(B)** Higher CD45 ^+-^infiltrated cells in the kidneys of CsA, SB203580, and SB202190 were detected. **(C)** Monocytic and lymphocytic cell compartments in the blood show a slight increase in cell number in animals treated with CsA and p38 MAPK inhibitors. B and C were measured by flow cytometry.

RNAseq revealed that mice treated with CsA or the p38 MAPK inhibitor show a similar increased gene expression. Gpnmb, Fgb, Vcam1, and Gc are genes indicating ongoing inflammation (Nguyen and Simpson-Haidaris, 2000; Kong et al., 2018; Saade et al., 2021) and are upregulated in these mice. Also, kidney-related genes such as Mgp, Ugt1a1, Abcb1b, and Angptl4 are showing an increased gene expression (Yamakawa et al., 2004; Miyata et al., 2018) (Figure 3A). Meanwhile, animals treated with VIVIT showed no such signatures. Moreover, immune cell infiltration (CD45^+^) into the kidney is upregulated in animals treated with CsA or p38 MAPK inhibitors (Figure 3B). Not only could we detect changes in kidney infiltrated cells, but immune cell compartments, notably CD45^+^ and CD11b + cells, seem to be increased upon CNI and slightly decreased under p38 MAPK inhibition (Figure 3C).

## Discussion

CNIs play a crucial role in effective immunosuppression after kidney transplantation. However, long-term treatment leads to chronic nephrotoxicity, known as calcineurin inhibitor toxicity. CsA and FK506 interfere with the cytosolic phosphatase calcineurin in lymphoid cells, preventing dephosphorylation NFAT and gene transcription. To date, it is unclear if the biological effects of CNI in non-lymphoid cells result similarly from disrupted calcineurin-NFAT signaling. This work aimed to investigate off-targets of CNI in epithelial cells. We present experimental evidence that CNT lesions are induced in kidney epithelial cells *in vitro* and *in vivo* independent of canonical calcineurin/NFAT signaling yet induced by kinase inhibitors, including inhibitors of the p38 MAPK, phosphoinositide-3-kinase (PI3K)/Akt, protein kinase C (PKC), and the inhibitor of nuclear factor-*κ*B (I*κ*B) kinase (IKK) pathway.

We showed that Fn14 upregulation is achieved by explicitly treating various kidney epithelial cells as well as *in vivo* with CNIs (CsA and FK506). Other compounds, known to be also nephrotoxic, however, failed to upregulate Fn14. These results confirm previous studies (Claus et al., 2018) that Fn14 is specifically upregulated due to calcineurin inhibitor toxicity in kidney epithelial cells. Therefore, the Fn14 is an excellent surrogate marker to measure nephrotoxicity and pro-fibrotic signals induced by CNIs.

Furthermore, we showed that an effective inhibitor (11R-VIVIT) of the NFAT axis fails to induce Fn14 in epithelial cells. When inhibiting the calmodulin kinase II, which is upstream of NFAT, no induction of Fn14 was detected. These results show that the direct NFAT inhibitor cannot mimic CsA’s activity in kidney epithelial cells. Therefore, nephrotoxic CNI signaling in kidney epithelial cells is not mediated *via* the canonical NFAT pathway *in vitro* and *in vivo*.

Likely, CNT is the consequence of the off-target effects of CsA in kidney epithelial cells. It has been hypothesized that CsA targets disparate signaling pathways in lymphoid and non-lymphoid cells (Berzal et al., 2012). We showed that CsA’s fibrotic effect on kidney cells is stable and easy to measure by the surrogate marker Fn14 and that the Fn14 expression is only upregulated under CNI treatment. As the nephrotoxic effect is NFAT-independent, we show here synthetic compounds and their capacity to induce Fn14. Our screen reveals that protein inhibitors of the serine/threonine p38 MAPK, PI3K/Akt, PKC, and IKK kinases mimic CsA’s activity on epithelial cells. These effects are specific for respective pathways since chemically unrelated inhibitors of these routes elicited similar activities (SB203580 and SB202190/wortmannin and triciribine/hypericin, GF 109203X, and palmitoyl-dl-carnitine). Meanwhile, inhibitors of MEK1/2-ERK (PD-98059/U-0126) or tyrosine kinases such as JAK2 and EGFRK (AG-490 and Tyrphostin 23) had no Fn14-inducing activities. Results strongly suggest that the CNT of CsA on kidney epithelial cells acts by inhibiting p38 MAPK and PI3K/Akt kinases. Both kinase pathways p38 MAPK and PI3K/Akt are important in epithelial cells for differentiation, proliferation and apoptosis, and extracellular matrix synthesis. In both pathways, the serine/threonine kinase GSK3⍺ is involved (Jacobs et al., 2012). Our top-down approach reveals that the inhibition of GSK3⍺ (by Indirubin-3′-monooxime) shows slight upregulation of Fn14. Lastly, CsA and the p38 MAPK inhibitor induced *in vitro* and *in vivo* nephrotoxic, inflammatory, and fibrotic genes. The p38 MAPK inhibitor upregulates similar genes as CsA, which supports that CsA acts on p38 MAPK as an off-target.

This work has several limitations. First, induction of sustained CNT lesions is cumbersome in inbred mice, and treatments require high supramaximal CsA doses. The reason for this notion is unknown yet likely lies in a greater renal reserve for hemodynamic toxicity, low-renin levels, and other factors (Thomas et al., 1994; Rabe and Schaefer, 2016; Karolin et al., 2021). Possibly, single nucleotide polymorphisms further contribute to this CsA resistance in inbred mice. CNT lesions are not highly specific to CsA or Tac exposure in mice and humans. Similar lesions, including arteriohyalinosis and interstitial fibrosis/tubular atrophy (IFTA), can be identified in patients with chronic active and chronic inactive rejection and kidney transplants from older and hypertensive donors (Myers et al., 1984; Einecke et al., 2017).

This work offers potential for future experimental and clinical projects. First, numerous nephroprotective treatments are available or awaiting clinical approval, including SGLT-2 inhibitors (Vogt, 2021), PLG1-analog (Marso et al., 2016), and finerenone (Bakris et al., 2020). Since CNT is likely an off-target effect of CsA directly on kidney epithelial cells, such nephroprotective drugs could be of great value to prevent/treat developing or existing CNT in solid organ transplant recipients. Second, it remains unknown which subpopulation of kidney cells is affected by CNI treatment. Therefore, the following experiments could be focused on which kidney cell types (i.e., tubules and podocytes) are affected by the nephrotoxic potential of CNIs.

CNT shows a highly variable clinical presentation, and some patients develop early and progressive lesions within months of SOT. In contrast, others tolerate similar CNT doses/through levels over many years or decades without significant graft or kidney function impairment and absence of CNT lesions in biopsy specimens (Baan et al., 2000; Karolin et al., 2021; Xia et al., 2018). The reason for this highly inter-individual predisposition is unclear but likely lies in single nucleotide polymorphisms (from the donor in a setting of kidney transplantation and the recipient). No GWAS study has yet been performed to decipher risk genes for rapid and progressive CNT after SOT.

In conclusion, these results suggest that CsA acts NFAT-independent on kidney cells yet requires p38 MAP and PI3K/Akt kinase pathways. Furthermore, we could show *in vitro* and *in vivo* that inhibition of proposed pathways (p38 MAPK and PI3K/Akt) mimics the toxicity of CsA on epithelial cells. However, we cannot exclude that CNT is not a highly specific interaction with a single molecular pathway.

## Data Availability

The datasets presented in this study can be found in online repositories. The names of the repository/repositories and accession number(s) can be found below: https://www.ncbi.nlm.nih.gov/geo/, GSE185146 https://www.ncbi.nlm.nih.gov/sra/PRJNA767789, PRJNA767789.
